# GFP Affects Human T Cell Activation and Cytokine Production following *In Vitro* Stimulation

**DOI:** 10.1371/journal.pone.0050068

**Published:** 2013-04-05

**Authors:** Kristi A. Koelsch, YuJing Wang, Jacen S. Maier-Moore, Amr H. Sawalha, Jonathan D. Wren

**Affiliations:** 1 Oklahoma Medical Research Foundation, Arthritis and Clinical Immunology Program, Oklahoma City, Oklahoma, United States of America; 2 University of Oklahoma Health Sciences Center, Department of Medicine, Oklahoma City, Oklahoma, United States of America; 3 Division of Rheumatology, Department of Internal Medicine, University of Michigan, Ann Arbor, Michigan, United States of America; 4 University of Oklahoma Health Sciences Center, Department of Biochemistry and Molecular Biology, Oklahoma City, Oklahoma, United States of America; Carl-Gustav Carus Technical University-Dresden, Germany

## Abstract

There are many Green Fluorescent Proteins (GFPs) originating from diverse species that are invaluable to cell biologists today because of their ability to provide experimental visualization of protein expression. Since their initial discovery, they have been modified and improved to provide more stable variants with emission ranges spanning a wide array of colors. Due to their ease of expression both *in-vitro* and *in-vivo*, they are an attractive choice for use as markers in molecular biology. GFPs are generally assumed to have negligible effects on the cells to which they have been introduced. However, a growing number of reports indicate that this is not always the case. Consequently, because of GFP's ubiquitous use, it is important to document the nature and extent of unintended effects. In this report, we find that GFP affects T cell activation, leading to defects in clustering, upregulation of the activation marker CD25 and IL-2 cytokine production following stimulation in human primary T cells that also express TurboGFP. We utilized a reporter assay which has been routinely used to assay the NF-κB pathway and found reduced NF-κB activitation in stimulated HEK293 and HeLa cells that were co-transfected with TurboGFP, suggesting that GFP interferes with signaling through the NF-κB pathway. These findings indicate that the utilization of GFP-tagged vectors may negatively impact *in vitro* experiments in T cells, emphasizing the critical importance of controls to identify any GFP-induced effects.

## Introduction

Green fluorescent proteins (GFPs) and their derivatives have rapidly become one of the most widely utilized protein families in cell biology. The first GFP was isolated from the jellyfish, *Aequorea victoria*, and has been subsequently modified to produce brighter and more photo-stable variants emitting in the blue, cyan and yellow spectral regions [1]. Red fluorescent proteins were later isolated from various species of the coral reef *Anthozoan* class, adding to the available color spectra [2]. Although currently available fluorescent proteins (FPs) originate from different species, have different protein sequences and emit in different spectral ranges, they share a common three-dimensional structure consisting of an 11-stranded β-barrel cylinder surrounding a central chromophore that is responsible for their fluorescent properties [3]–[7].

Due to the wide range of colors, increased protein stability, and ease of expression, FPs have gained popularity as markers in molecular biology. FPs have also become an indispensable tool to follow transfection efficiencies, a critical feature of some applications such as RNA interference (RNAi) gene knockdown, where low transfection efficiencies could potentially mask any resulting phenotypic changes. One example of these is TurboGFP, a derivative variant of the copGFP cloned from the copepod, *Pontellina plumata*. TurboGFP is used in many transfection vector constructs as a constitutively expressed protein enabling rapid identification of transfected cells by either fluorescence microscopy or flow cytometric analysis. In primary cells and in cell lines where transfection efficiencies are inherently low, GFP-positive flow cytometric cell sorting can effectively increase transfection efficiency to nearly 100 percent.

The use of FPs as endogenously produced protein markers is generally thought to have negligible effects on cellular function. However, a growing number of reports indicate that FP effects on cellular functions may not be as innocuous in all systems as previously believed [8]–[14]. We happened upon this phenomenon in a series of RNAi experiments designed to identify genes with roles in primary human T cell activation using a TurboGFP-tagged shRNA knockdown vector. We observed defects of activation in primary T cells transfected with GFP vectors containing either gene-specific or scrambled (control) shRNA. These defects were not seen with non-GFP control vectors or non-labeled siRNAs. Therefore, we hypothesized that TurboGFP may have inhibitory effects on the activation of primary T cells and sought to explore and characterize the observed effects. Herein, we demonstrate that TurboGFP inhibits not only human primary T cell activation pathways, but also downstream cytokine production. We demonstrate that TurboGFP-tagged vectors negatively impact experiments where assessment of T cell activation-dependent markers and cytokine production are the final outcome.

## Materials and Methods

### T Cell Isolation, Transfection and Activation

PBMC's were freshly isolated from healthy donor buffy coats (Oklahoma Blood Institute, Oklahoma City, Oklahoma) by density gradient centrifugation (Ficoll-Paque Plus, G.E. Healthcare, Piscataway, NJ, USA) using the manufacturer's protocol. T cells were then isolated by negative selection from the PBMC's using the MACS Pan T Cell Isolation Kit II (Miltenyi Biotec, Inc., Auburn, CA, USA). T cell isolation efficiency routinely resulted in >98% purity as measured by flow cytometric analysis.

Plasmid constructs (pGFP-V-RS and pRS) were obtained from Origene Technologies (Rockville, MD, USA). The constructs utilize the same vector backbone and scrambled (non-effective) shRNA sequence, with the GFP(+) version (pGFP-V-RS #TR30013) having a CMV-driven GFP cassette which is absent in the GFP(−) version (pRS #TR30012). Each plasmid contained a antibiotic resistance marker (Kanamycin/Puromycin and Ampicillin, respectively). 1×10^7^ purified T cells were transfected with 2.5 µg of either shRNA vector using the Human T Cell Nucleofector Kit (#VPA-1002, Lonza, Cologne, Germany). In some experiments the unlabeled scrambled vector (Scr-Vec-GFP(−) was directly labeled with fluorescein (FITC) using the LabelIT Tracker Nucleic Acid Labeling Kit (#MIR3225, Mirus Bio, Madison, WI, USA) to positively identify transfected cells. Transfected T cells were left to rest overnight in complete T cell media (cTCM; DMEM containing 10% FCS, NEAA, Sodium Pyruvate, β-ME (50 µM), followed by a cTCM medium change 12 hours post-transfection. Our transfection efficiency is consistently 40–50% by this method when using the shRNA vectors.

Approximately 48–60 hours post-transfection, T cells were washed with cTCM and activated for 20 hours by plating into 12-well tissue culture plates coated with 10 µg/ml anti-CD3 (clone OKT3, #16-0037-85, eBiosciences, San Diego, CA, USA), in cTCM containing 2.5ug/ml anti-CD28 (Clone CD28.2, #16-0289-85, eBiosciences, San Diego, CA, USA). After stimulation, cells were visualized by fluorescent microscopy, supernatants collected for the analysis of IL-2 by ELISA and the cells harvested for flow cytometric and real-time PCR analysis.

### Activation analysis by Microscopy and Flow Cytometry

Cultures were visualized for clustering 20 hours following activation using a Zeiss Axiovert 200 M inverted microscope (Carl Zeiss, Inc., Thornwood, NY, USA) at the OMRF Imaging Core Facility. Images were taken using an AxioCam HRm Rev. 3 color camera (Carl Zeiss Microscopy, Thornwood, NY, USA) at 100X magnification. T cells were stained for sorting and flow cytometric analysis with anti-CD4-APC (#300514, BioLegend, San Diego, CA, USA), anti-CD8-PerCP-Cy5.5 (#344710, BioLegend, San Diego, CA, USA), anti-CD25-V450 (#560355, BD Biosciences, San Jose, CA, USA), and anti-CD 69-APC-Cy7 (#310914, BioLegend, San Diego, CA, USA). CD3/CD28-activated T cells were bulk sorted for RNA analysis into CD4^+^ and CD8^+^ populations using a Becton-Dickinson FACSAria Cytometer (BD Biosciences, San Jose, CA, USA) and each population was then analyzed for expression of the activation markers CD25 and CD69 using FlowJo flow cytometry analysis software (Tree Star, Inc., Ashland, OR, USA).

### RNA Isolation and Real-Time PCR

RNA was isolated from bulk-purified T cells using an RNeasy RNA Isolation Kit (Qiagen, Valencia, CA, USA) according the manufacturer's suggested protocol with modifications. Briefly, sorted cells were pelleted in 1.5 ml microcentrifuge tubes, lysed in 1 ml Trizol (Invitrogen, Carlsbad, CA, USA) then frozen at −70°C overnight. Following thawing and addition of 200 µl of chloroform, lysates were mixed by inversion for 15 s, incubated at RT for 3 min and centrifuged at 4°C and 14,000 RPM in an Eppendorf 5415R Microcentrifuge for 15 min. The aqueous layer was transferred into a new tube to which 0.5× volumes of 100% ethanol was added, mixed by pipetting and applied to the RNeasy Kit columns. The columns were washed and RNA eluted with 50ul of DNase- and RNase-free water. Contaminating DNA was removed using the Turbo DNA-Free Kit (Ambion, Austin, TX, USA). RNA was quantified by Nanodrop-1000 spectrophotometric analysis (Thermo Scientific, Wilmington, DE, USA). Primer sequences were as follows: CD25 forward, 5′-ATCAGTGCGTCCAGGGATAC-3′, CD25 reverse, 5′GACGAGGCAGGAAGTCTCAC-3′, ACTB forward, 5′ GCACCACACCTTCTACAATGAGC -, and ACTB reverse, 5′-GGATAGCACAGCCTGGATAGCAAC. Primers were purchased from Integrated DNA Technologies (Coralville, IA, USA). Reverse transcription and real-time PCR reactions were performed for CD25 expression in 20 µl using 25 ng of RNA and CD25-specific primers (300 nM) with the iScript One-Step RT-PCR kit containing Sybr Green (Bio-Rad, Hercules, CA, USA) according to the manufacturer's protocol. All samples were normalized to the control housekeeping gene β-Actin (ACTB). Relative expression levels were calculated using the 2-(delta-delta (CT)) method [15].

### IL-2 ELISA

IL-2 cytokine concentrations were measured using a Human IL-2 Colorimetric ELISA kit (Pierce/Thermo Fisher Scientific, Rockford, IL, USA) according to the manufacturer's protocol. A standard curve was generated using serial dilutions of the IL-2 standard to calculate the concentrations of unknown samples. All samples were measured in duplicate and corrected for non-specific and secondary background binding. To normalize for variability between donors each donor's Scr-Vec-GFP(+)-transfected sample was normalized to its respective Scr-Vec-GFP(−)-transfected sample. Readings were made using a BioTek EL800 Microplate reader and Gen5 data analysis software (Winooski, VT, USA).

### Luciferase Assays

HEK-293A (HEK) and HeLa cells, kindly provided by the OMRF Human Antibody Core Facility and Dr. Gary Gorbsky, respectively, were cultured in Iscove's Modified Dulbecco's Medium supplemented with 10% FBS (IMDM-FBS) and seeded in 24-well plates at a density of 1.5×10^ 5^ cells per well (HEK) or 5×10^4^ cells per well (HeLa) 24 hours prior to transfection. Either the Scr-Vec-GFP(+) or Scr-Vec-GFP(−) vector (100 ng) was co-transfected with luciferase-tagged NF-κB and renilla constructs into cells using linear Polyethylenimine (PEI #23966; Polysciences Inc., Warrington, PA, USA) as previously described [16]. Briefly, immediately prior to transfection, media was changed to IMDM containing 1% FBS. For each transfection, DNA was incubated with 1 µl PEI solution in 50 µl IMDM media lacking FBS for 15 min at RT, then added drop-wise to the cells and incubated overnight at 37°C. Following incubation, the media was replaced with IMDM-FBS followed by activation with PMA (10 ng/ml) and ionomycin (400 ng/ml) for 16 hours. Cells were harvested and the expression of NF-κB and renilla reporters was assessed using the Dual-Glo Luciferase Assay System (Promega Corp., Madison, WI, USA) and read on a Biotek Synergy HT plate reader (Biotek, Inc., Winooski, VT, USA) using Gen5 software (Biotek, Inc., Winooski, VT, USA) according to the manufacturer's protocols. Transfections and luminescence readings were evaluated in triplicate. For data analysis, all samples were corrected for background fluorescence and luciferase activity was calculated as a ratio of luminescence units for luciferase:renilla for each sample.

## Results

### T Cells Transfected with TurboGFP Display Defects in Clustering Following Activation

**Figure 1:**
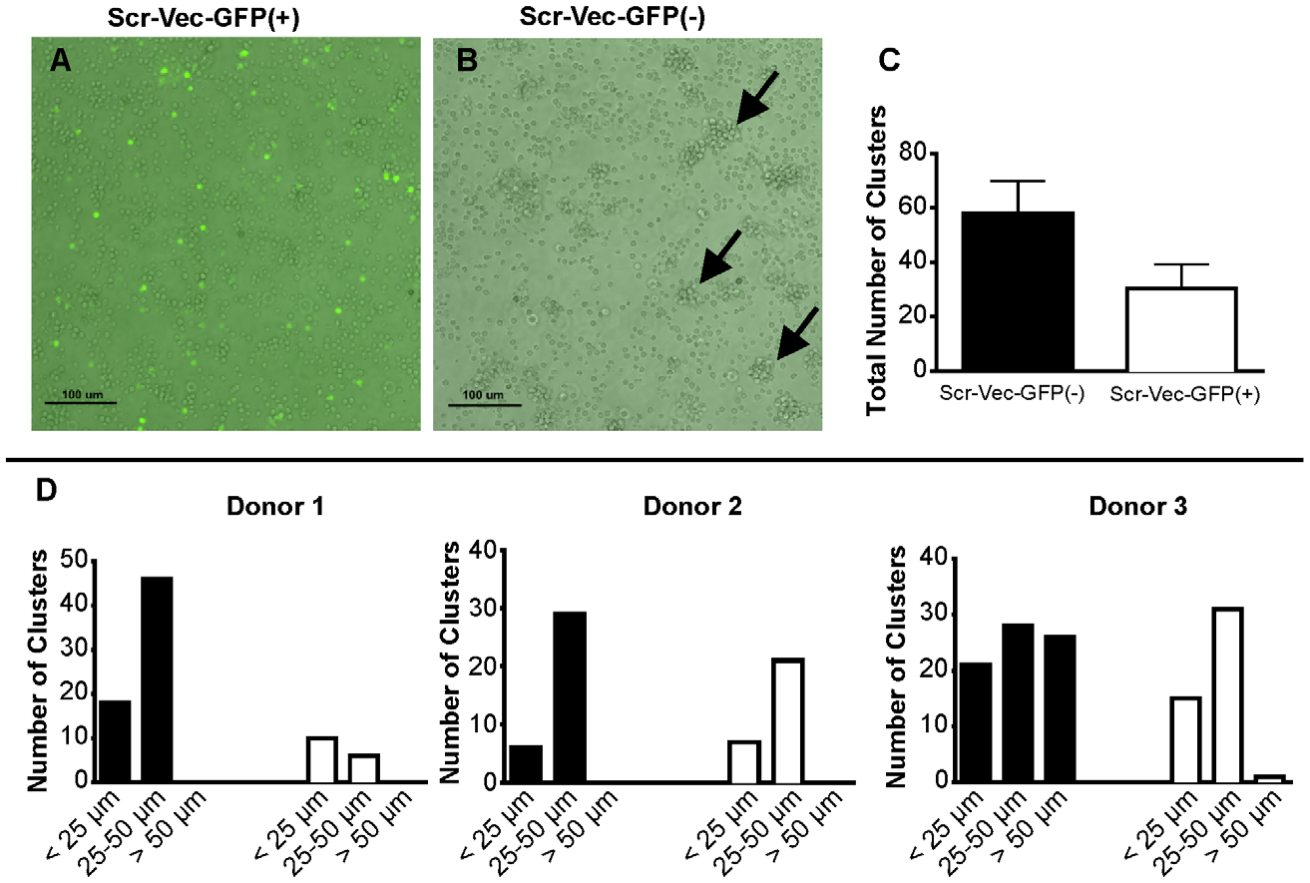
TurboGFP impairs T cell clustering after CD3/CD28 activation. T cells were transfected with either the Scr-Vec-GFP(+) or the Scr-Vec-GFP(−) vectors, then activated by plate-bound anti-CD3 and soluble CD28 for 20 hours. A) GFP-expressing T cells exibit impaired clustering after CD3/CD28 activation. B) Normal cluster formation of CD3/CD28-activated T cells in sample transfected with the Scr-Vec-GFP(−) are indicated by black arrows. A representation of 3 independent experiments is shown. C & D) Activated cell clusters were quantified within one representative field of view at 100x for the Scr-Vec-GFP(−) vector and the Scr-Vec-GFP(+) vector transfections to obtain the total number of clusters (C), or the numbers of clusters by size range (<25 μm, 25–50 μm, and >50 μm) (D) where the black bars indicate the Scr-Vec-GFP(−) transfections and the white bars indicate the Scr-Vec-GFP(+) transfections. Mean and SEM are shown.

We observed defects in the characteristic clustering of activated T cells transfected with a scrambled (non-specific) control shRNA vector containing the TurboGFP cassette (Scr-Vec-GFP(+); Fig. 1A). Conversely, normal clustering was observed in T cells transfected with the scrambled control shRNA vector lacking GFP expression (Scr-Vec-GFP(−); Fig. 1B). A trend toward increased numbers of clusters (Fig. 1C) and an increase in cluster size was observed in the transfections lacking GFP (Scr-Vec-GFP(−); Fig. 1D), suggesting that the presence of GFP impairs the ability of T cells to cluster following activation.

### TurboGFP+ T Cells Display Defects in Surface CD25 but not CD69 Expression in Primary T Cells Following Activation

**Figure 2:**
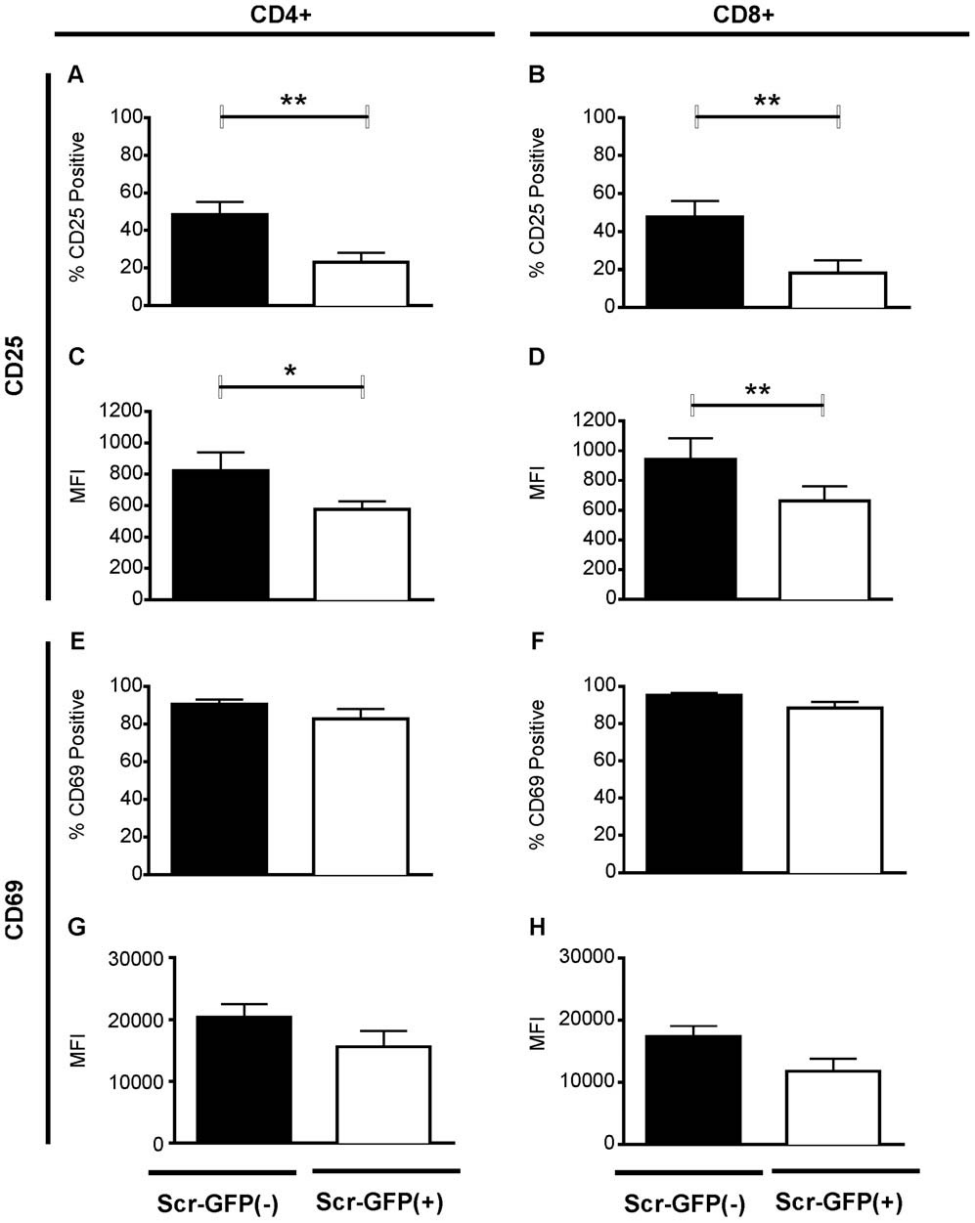
Analysis of CD25 and CD69 surface expression by flow cytometry. T cells from seven donors were transfected with the vectors Scr-Vec-GFP(−) (black bars) or Scr-Vec-GFP(+) (white bars). Following 20 hours of anti-CD3/anti-CD28 activation, CD4^+^ and CD8^+^ populations were analyzed for CD25^+^ expression and compared by Student's paired t-test in the CD4^+^ (A) and CD8^+^ (B) populations (*P* = 0.006 and *P* = 0.04, respectively). Mean Fluorescence Intensity (MFI) for CD25 expression in CD4^+^ (C) and CD8^+^ (D) populations indicate significant differences for CD4^+^ populations (*P* = 0.02) and CD8^+^ populations (*P* = 0.003). E–H) CD69 expression analysis indicates no significant differences in either proportion of CD4^+^ or CD8^+^ CD69-expressing cells or in the MFIs. Mean and SEM are shown and *P*≤0.05 was considered to be significant.

It is well known that CD25 and CD69 are both upregulated following T cell activation and are classical markers used to determine activation state. We used flow cytometric analysis to determine the level of activation in primary peripheral blood T cells from seven healthy donors transfected with Scr-Vec-GFP(+) or Scr-Vec-GFP(−) shRNAs following CD3/CD28 stimulation. In those samples transfected with the Scr-Vec-GFP(+) compared to the Scr-Vec-GFP(−) vector vector, we observed a significant decrease not only in the percentage of CD25^+^ cells in both CD4^+^ (Fig. 2A; P = 0.007) and CD8^+^ (Fig. 2B; P = 0.004) populations, but also in CD25 mean surface receptor density assessed as mean fluorescence intensity (MFI) for CD4^+^ (Fig. 2C; P = 0.02) and CD8^+^ (Fig. 2D; P = 0.003) cell populations. No significant differences were observed for CD69 in regard to either the percentage of CD4^+^ or CD8^+^ T cells expressing the activation marker (CD4^+^, *P* = 0.07 and CD8^+^, *P* = 0.06) or in the MFI (CD4^+^, *P = *0.10 and CD8^+^, *P* = 0.07) (Figs. 2E–H), suggesting a CD25 pathway-specific inhibition of T cell activation.

**Figure 3:**
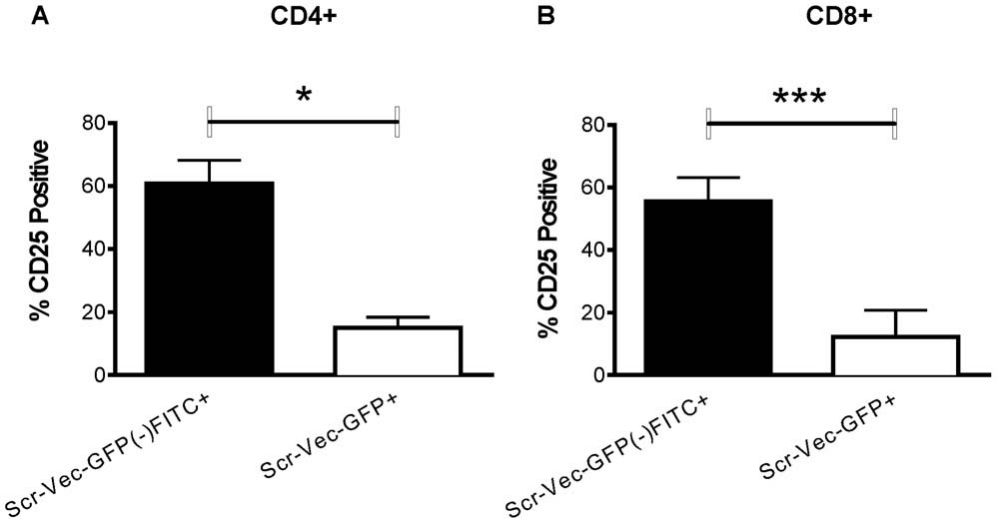
CD25 surface expression by flow cytometry in FITC-labeled Scr-Vec-GFP(−) and Scr-Vec-GFP(+) cells. CD25 surface expression was measured by flow cytometry. T cells from three donors were transfected with the Scr-Vec-GFP(−) directly labeled with FITC (black bars) or Scr-Vec-GFP(+) (white bars). Following 20 hours CD3/CD28 activation cells were stained for flow cytometry analysis. Cells were gated on the total live T cell population by forward and side-scatter analysis, followed by gating on either FITC+ or GFP+ cells, then CD4^+^ (A) or CD8^+^ (B) populations were analyzed for CD25 expression and compared by Student's paired t-test. Mean and SEM are shown and *P*≤0.05 was considered to be significant.

It is conceivable that these differences were caused by differences in transfection efficiencies. While it is generally accepted that nearly identical vectors would transfect with approximately the same efficiency, our experimental design made it difficult to measure transfection efficiencies in the Scr-Vec-GFP(−) transfections. Therefore, we used flow cytometry to include only those cells known to be transfected in the analysis. To accomplish this, we directly labeled the Scr-Vec-GFP(−) vector with fluorescein isothiocyanate (FITC). As expected, the the CD4^+^ (Fig. 3A) and CD8^+^ (Fig. 3B) populations transfected with the Scr-Vec-GFP(+) vector showed decreased CD25 expression when compared to CD4^+^ and CD8^+^ populations transfected with the FITC-conjugated Scr-Vec-GFP(−) vector (CD4^+^, *P* = 0.01, and CD8^+^, *P* = 0.0006, n = 3).

### CD25 mRNA Transcript Levels are Decreased in GFP+ Activated T Cells compared to GFP- Activated T cells

**Figure 4:**
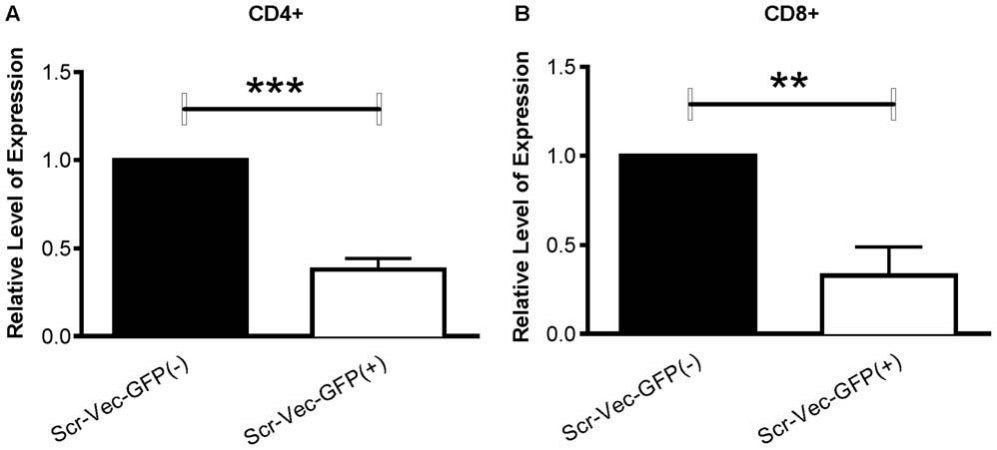
CD25 expression levels by RT-PCR. RNA was isolated from CD4^+^ (A) and CD8^+^ (B) populations from six donors following 20 hours activation with anti-CD3/anti-CD28. Relative CD25 expression levels were normalized to beta-actin, calculated using the 2-(delta-delta(CT)) method and compared by Student's paired t-test. Mean and SEM are shown and *P*≤0.05 was considered to be significant.

To determine whether the decrease in CD25 cell surface expression of the cells transfected with Scr-Vec-GFP(+) also occurred at the mRNA transcript level, we measured CD25 mRNA expression levels in CD4^+^ and CD8^+^ populations from six of the donors for which surface analyses were made by real-time PCR (RT-PCR). Consistent with the flow cytometric data, CD25 expression levels were significantly reduced in both the CD4^+^ (Fig. 4A, *P* = 0.0002) and the CD8^+^ (Fig. 4B, *P* = 0.009) populations transfected with the Scr-Vec-GFP(+) relative to the Scr-Vec-GFP(−) vector.

### IL-2 Secretion is Decreased in GFP+ Compared to GFP- T Cells Upon Activation

**Figure 5:**
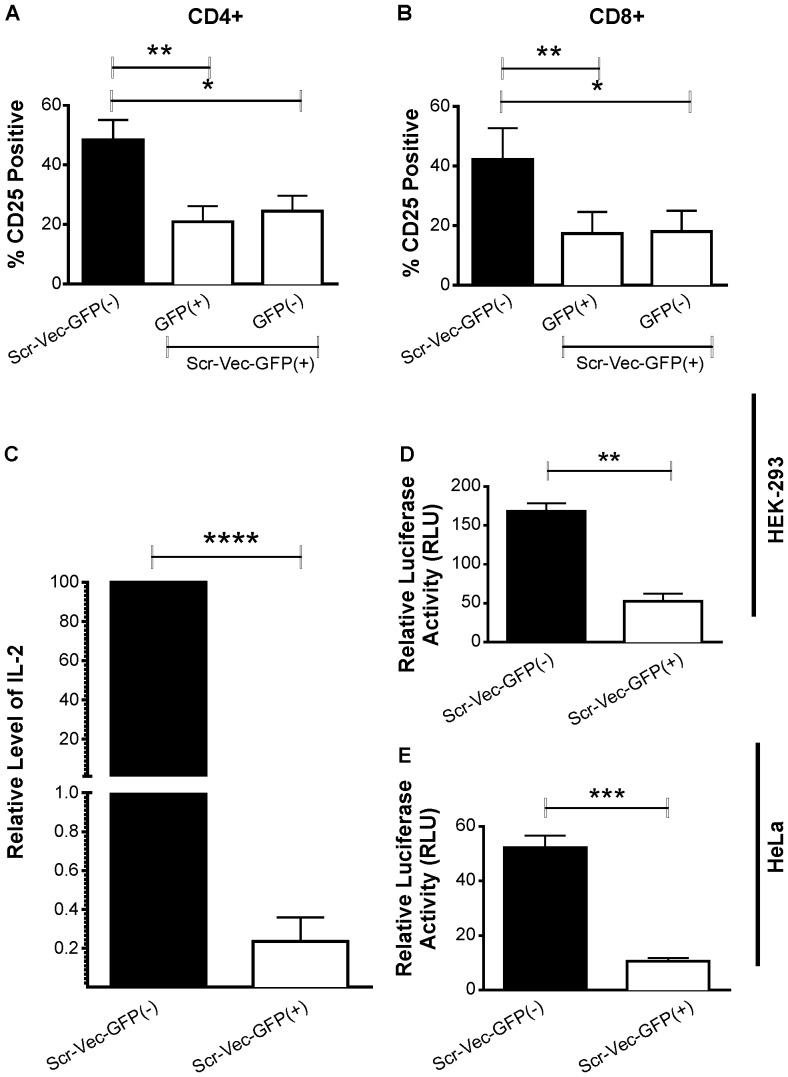
CD25 analysis of GFP(+) vs. GFP(−) populations within the Scr-Vec-GFP(+) vector transfections, IL-2 secretion by IL-2 ELISA, and NF-κB luciferase reporter assays. A & B) CD25 surface expression analysis in CD4^+^ (panel A) and CD8^+^ T cells (panel B) from 7 donors by flow cytometry comparing the Scr-Vec-GFP(−)-transfected cells to both the GFP+ (transfected) and GFP- (untransfected) portions of the Scr-Vec-GFP(+)-transfected cells indicates both the transfected (GFP+) and untransfected (GFP-) fractions of the Scr-Vec-GFP+ vector transfections are similarly and significantly lower than the Scr-Vec-GFP- vector transfected cells. C) IL-2 in the supernatant of T cells from six donors transfected with Scr-Vec-GFP(−) or Scr-Vec-GFP(+) vectors following 20 hours activation by anti-CD3/anti-CD28 was measured in duplicate by ELISA. D & E). HEK-293 cells (D) and HeLa cells (E) were co-transfected with NF-kB-luciferase, constitutively-expressed renilla and either the Scr-Vec-GFP(−) or Scr-Vec-GFP(+) vectors, then stimulated with PMA and ionomycin for 16 hours. Relative luciferase activity (luciferase:renilla) was assessed in triplicate for each transfection. Mean and SEM are shown and *P*≤0.05 was considered to be significant by Student's paired t-test.

We have observed that transfection efficiencies for human primary T cells consistently range between 40–50% using nucleofection-based methods. We noted that the reduction in CD25 surface protein expression observed in the Scr-Vec-GFP(+) transfection reactions was not limited to the transfected (GFP-positive) cells within the culture. We observed that the untransfected (GFP-negative) cells in these reactions also demonstrated significant decreases in CD25 levels when compared to the Scr-Vec-GFP(−) transfection reactions (Figs. 5A and 5B; CD4+ Scr-Vec-GFP(+) populations, transfected *P* = 0.003 vs. untransfected *P* = 0.01; CD8+ Scr-Vec-GFP(+) populations, transfected *P* = 0.006 vs. untransfected *P* = 0.03). This suggested that a soluble factor secreted in the culture media might be involved. IL-2 was a logical candidate as it acts in both an autocrine and paracrine manner on its high affinity receptor, CD25. A reduction in IL-2 secretion from a large portion of GFP-expressing cells could lower the overall IL-2 concentration in the cell culture supernantant, which has been demonstrated by others to directly affect the expression levels of CD25 on stimulated T cells *in vivo* [17], [18]. Supernatants of T cell cultures isolated from six different donors stimulated with CD3/CD28 and transfected with Scr-Vec-GFP(+) or Scr-Vec-GFP(−) were assessed for IL-2 production by sandwich-ELISA. Consistent with our hypothesis, IL-2 levels in the supernatant were significantly reduced in the cultures following transfection with the Scr-Vec-GFP(+) but not with the Scr-Vec-GFP(−) vector (Fig. 5C, *P* = 0.0001).

### GFP inhibits NF-κB Activity in HEK-293 and HeLa Reporter Assays

Since NF-κB activation is critical for T cell activation and for driving IL-2 and CD25 expression, we hypothesized that decreased NF-κB activation in the Scr-Vec-GFP(+) transfectants could explain the reduced IL-2 and CD25 expression in these cells. To test this, we utilized NF-κB-luciferase/renilla HEK-293 and HeLa cell line reporter assays to measure NF-κB activity in Scr-Vec-GFP(+)- versus Scr-Vec-GFP(−)-transfected cells following stimulation with PMA and ionomycin, inducers of the NF-κB pathway. A significant decrease in NF-κB activity was observed in cells expressing GFP compared to those without in both the HEK-293 (*P* = 0.001; n = 3) and the HeLa (*P* = 0.0008; n = 3) cell lines (Fig. 5D and 5E, respectively).

## Discussion

GFP is generally presumed to be biologically inactive and to serve as a reporter to determine the effectiveness of molecular manipulations on cell populations, including detection of transfected genetic materials into cells or tagging of cloned proteins. The utilization of GFP as a marker in cell biology is widespread and has proven to be a indispensable tool in many biological studies. However, should the presence of GFP exert a measurable biological effect upon cellular activity or activation state within a cell, it can compromise assay interpretation and/or validity. Indeed, increasing numbers of reports have shown the negative influences of GFP expression on various systems [8]–[14].

We evaluated this in our experimental systems utilizing GFP-labeled and unlabeled versions of a scrambled shRNA vector tested side-by-side. Following transfection and activation of human primary T cells, we compared expression levels of T cell activation markers, CD25 and CD69. Significant decreases in CD25 expression at both the mRNA and surface protein levels were observed in both the CD4^+^ and CD8^+^ populations following transfection with the GFP+ vector. CD69 is a very early activation marker that can be upregulated by the NF-κB activation pathway, however it can also be regulated by other, non-canonical activation pathways [19]. We did not observe defects in cell surface upregulation of CD69 in the GFP(+) transfected samples. This indicates that the decreased CD25 expression that we observe in the GFP(+) transfected samples is an effect specific to the CD25 regulation pathway, as CD69 responds normally to the CD3/CD28 stimulation.

NF-κB activation, which was significantly reduced in HEK and HeLa cells transfected with the Scr-Vec-GFP(+) vector, is essential to T cell activation and subsequent upregulation of IL2 and CD25 (IL2R) [20], [21]. Expression of IL-2 is upregulated in activated T cells and helps to drive the upregulation of its receptor, CD25 (IL2R), by a feedback loop acting in both an autocrine and a very efficient paracrine fashion [22]. The reduction in IL-2 secretion we observed in cells transfected with the GFP-expressing vector could therefore account for the reduced CD25 expression in untransfected cells within the culture. In support, others have demonstrated that reduced IL-2 levels in cell culture supernatants reduces CD25 expression and activation levels in stimulated T cells [17], [18].

Thus, our data suggest that GFP may interact with one of the many mediators of the NF-κB pathway and thereby cause a reduction in IL-2 secretion and CD25 expression in activated T cells. Consistent with this hypothesis, the reduction in CD25 mRNA levels suggests that the decrease in CD25 surface protein expression observed by flow cytometry occurs at the transcriptional level, rather than by inhibition of CD25 protein trafficking to the cell surface. Interestingly, another group has reported a polyubiquitination defect attributed to GFP which results in reduced NF-κB activation in a reporter system using HEK-293T cells co-transfected with a NF-κB-luciferase reporter and an EGFP-expressing (Enhanced GFP from the species *Aequorea victoria*) vector [10].

Whether the *in vitro* effects of the GFP derivative described herein is specific to human T cells is unknown. Lai, et al. reported that *in vitro* murine T cell activation was not negatively affected by GFP, however, these experiments utilized EGFP and not TurboGFP [23]. Baen, et al. reported GFP-specific effects where EGFP negatively affected their experimental system, while another GFP derivative did not, demonstrating that GFP variants can affect the same system differently [10]. Negative effects have not only been reported to be GFP-specific, but also cell type specific within the same system [24]. Many transgenic mouse models have been developed utilizing numerous GFP-derivative variants *in vivo* as biomarkers for genetic manipulations. Transgenic models with endogenous GFP expression may undergo tolerogenic mechanisms, therefore, generalization or extrapolation of *in vitro* experimental results to *in vivo* experimental systems would not be appropriate.

## Conclusions

Together, these results demonstrate that transfection and expression of TurboGFP has a negative effect on T cell activation in both the CD4^+^ and CD8^+^ populations in our system. We have demonstrated that in activated T cells, TurboGFP can negatively affect IL-2 secretion and CD25 expression, both of which are critical to various pathways involving T cell growth and differentiation. We have also provided evidence that GFP expression may negatively impact NF-κB activity, which is critical to many immune cell and non-immune cell functional pathways. This study indicates that experimental designs incorporating expression of GFP in human T cells with subsequent CD3/CD28 activation may be negatively affected, further emphasizing the need for the appropriate transfection controls when utilizing GFP-expressing vectors.
